# Influence of Ultrasonic Activation of Endodontic Irrigants on Microbial Reduction and Postoperative Pain: A Scoping Review of In Vivo Studies

**DOI:** 10.3390/dj13100459

**Published:** 2025-10-08

**Authors:** Jacob Marx, Corban Ward, Bayler Gunnell, Zachary Marx, Alicia Parry, Samuel Dyal, Amir Mohajeri, Man Hung

**Affiliations:** 1 College of Dental Medicine, Roseman University of Health Sciences, South Jordan, UT 84095, USA; 2 Library, Roseman University of Health Sciences, South Jordan, UT 84095, USA; 3 Division of Public Health, University of Utah, Salt Lake City, UT 84108, USA; 4 Department of Education Psychology, University of Utah, Salt Lake City, UT 84112, USA; 5 VA Salt Lake City Health Care, Salt Lake City, UT 84148, USA

**Keywords:** endodontics, root canal, ultrasonic irrigation, acoustic streaming, cavitation, clinical outcomes

## Abstract

**Objective:** Root canal irrigation plays a critical role in achieving effective chemomechanical disinfection during endodontic therapy. Conventional syringe irrigation, typically using sodium hypochlorite, ethylenediaminetetraacetic acid, and chlorhexidine, is limited by its delivery method and often fails to adequately penetrate complex canal anatomies, compromising disinfection. Advancements such as ultrasonic and multisonic irrigation systems aim to address these limitations. This scoping review compares the clinical effectiveness of ultrasonic irrigation techniques with conventional syringe irrigation, focusing exclusively on in vivo studies conducted within the oral environment. **Methods:** A comprehensive scoping review was conducted using PubMed, Scopus, Dentistry & Oral Sciences Source, and Google Scholar. Peer-reviewed, full-text articles published in English between 2015 and 2025 were screened by four independent reviewers based on predefined inclusion and exclusion criteria. Eligible studies were thematically analyzed. **Results:** Of 312 records screened, eleven studies met the inclusion criteria. Ultrasonic irrigation was associated with improved clinical outcomes, particularly greater reductions in bacterial load and endotoxins; however, findings regarding its effect on postoperative pain were inconsistent, with some studies reporting a benefit while others observed no significant difference. These outcomes were attributed to mechanisms such as acoustic streaming and cavitation, which enhance irrigant penetration, promote fluid dynamics, and facilitate debridement in anatomically complex regions. **Conclusions:** Ultrasonic irrigation appears to hold promise for enhancing the efficacy and efficiency of root canal treatment. Existing in vivo studies suggest potential clinical advantages over conventional syringe irrigation, underscoring the need for further high-quality clinical research to more definitively establish its benefits.

## 1. Introduction

Root canal irrigation is a fundamental component of endodontic therapy, playing a pivotal role in the success of root canal treatment (RCT). RCT is a procedure aimed at eliminating infection and preserving the natural tooth by removing infected pulp tissue from the root canal system [1]. An effective irrigation technique is essential to the success of a RCT, as it facilitates the removal of debris, microorganisms, and organic tissue from the complex canal anatomy [2]. Irrigation solutions, delivered using specialized techniques, serve to disinfect the canal, dissolve tissue, and condition the dentin for subsequent obturation [3]. The efficacy of irrigation directly influences the long-term success of RCTs, as residual bacteria or debris can lead to persistent infection and treatment failure.

Conventional syringe irrigation methods primarily involve syringe-based delivery of chemical irrigants, such as sodium hypochlorite (NaOCl), ethylenediaminetetraacetic acid (EDTA), and chlorhexidine (CHX) [4]. NaOCl is widely used for its antimicrobial properties and ability to dissolve organic tissue. EDTA complements NaOCl by chelating calcium ions to remove the smear layer. CHX, with its broad-spectrum antimicrobial activity and substantivity, is often employed as a final irrigant, especially in cases of necrosis [5]. These irrigants, when delivered through syringe irrigation, aim to disinfect and clean the canal system; however, their effectiveness is highly dependent on the delivery technique and the ability to reach the intricate anatomical structures within the root canal [6].

Despite their widespread use, conventional syringe irrigation techniques face significant challenges. One of the main limitations of syringe irrigation is its tendency to contribute to vapor lock formation in the apical region, which can hinder effective irrigant delivery [7]. The passive delivery of irrigants often does not penetrate dentinal tubules, lateral canals, or apical ramifications, leaving portions of the canal system inadequately cleaned [4]. Additionally, there is a risk of apical extrusion of the irrigant, which can lead to postoperative pain or periapical tissue damage [8]. These limitations have driven the development of advanced irrigation technologies aimed at enhancing the efficacy and safety of root canal disinfection.

In response to these limitations, newer technologies such as GentleWave^®^ and ultrasonic irrigation have been utilized to enhance canal cleanliness and reduce bacterial load. Ultrasonic irrigation involves the activation of irrigating solutions, such as NaOCl or EDTA, using ultrasonic energy transmitted through a vibrating metal tip or file inserted into the canal [9,10]. This technique employs high-frequency sound waves to generate both acoustic streaming and cavitation effects [11]. Cavitation refers to the formation and collapse of microscopic bubbles, which produce shock waves that disrupt biofilms and dislodge debris [12]. Acoustic streaming, on the other hand, induces rapid fluid movement, thereby enhancing the penetration of the irrigant into complex anatomical structures such as lateral canals, isthmuses, and dentinal tubules [13].

While previous work has predominantly evaluated procedures conducted on extracted or bovine teeth [14,15], there remains a gap in understanding the clinical performance of these irrigation systems in vivo [16]. This scoping review aims to compare ultrasonic irrigation techniques with conventional syringe irrigation, focusing exclusively on studies conducted in vivo, within the oral environment. By synthesizing clinical evidence, this review seeks to provide insights into the effectiveness, safety, and applicability of these irrigation methods in real-world RCT scenarios, thereby guiding clinicians in optimizing endodontic outcomes. This review focuses on two primary outcomes when comparing ultrasonic and syringe irrigation: the effectiveness in reducing microbial load and the impact on post-operative pain.

## 2. Methods

This scoping review included peer-reviewed articles published in English from 2015 to 2025 that compared conventional irrigation with ultrasonic irrigation in human patients. The Preferred Reporting Items for Systemic Reviewers and Meta-Analyses and extensions for Scoping Reviews (PRISMA-ScR) guidelines were followed for this study [17]. Only full-text original research articles were included, while reviews (such as literature reviews, systematic reviews, and meta-analyses), editorials, letters to the editor, and conference abstracts were excluded. Studies published before 2015, not written in English, or not specifically focused on comparing irrigation efficacy between conventional methods and ultrasonic activation in endodontic therapy were also excluded to ensure the relevance and specificity of the review.

Major scientific databases—PubMed, Scopus, Dentistry & Oral Sciences Sources and Google—were comprehensively searched to identify all articles comparing ultrasonic irrigation to other forms of irrigation. The search approach integrated Medical Subject Headings (MeSH) and relevant keywords from article titles and abstracts, utilizing Boolean operators (AND, OR, NOT) to optimize search precision. Search terms included: root canal, pulpectomy, side vented needle, syringe, ultrasonic, GentleWave, conventional techniques, chemical irrigation, success, and clinical outcomes. (Table 1) All search results were managed using EndNote 21 software, which also facilitated the removal of duplicate entries.

Four independent reviewers (J.M., C.W., B.G., and Z.M.) screened the titles and abstracts of all identified records according to predefined inclusion and exclusion criteria (outlined in Table 2). Any disagreements were resolved through discussion or consultation with an additional reviewer (M.H.). During data extraction, standardized data collection forms were used to systematically gather essential information from each included study. This process was conducted independently by two reviewers, who recorded details such as study design, publication year, research aims, findings, and conclusions. Any conflicts in data extraction were resolved through consensus or by involving a third reviewer (M.H.). This methodology was designed to ensure accuracy and improve the overall reliability of the collected data.

## 3. Results

**Figure 1:**
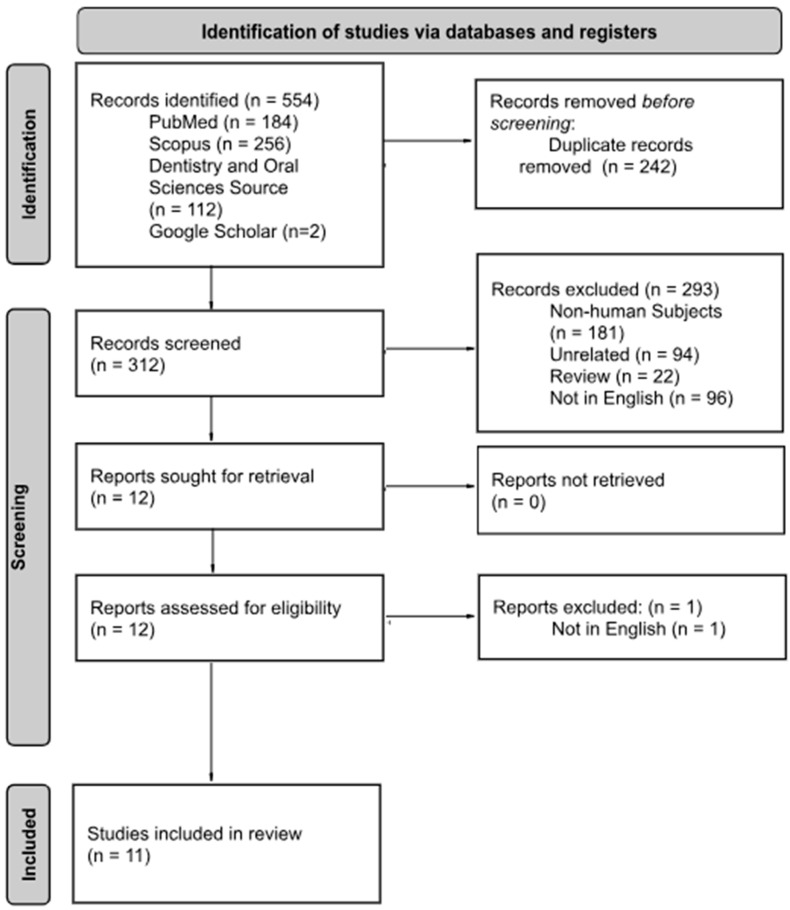
PRISMA Flow Diagram adapted from the PRISMA-ScR guidelines.

From the database search, a total of 552 articles were initially identified. Additionally, a search of Google Scholar was used to include two additional in vivo studies that fit the inclusion criteria making the total articles 554. Prior to screening, 242 duplicates were removed. The remaining 312 articles were screened by the four independent reviewers using the aforementioned inclusion and exclusion criteria. After the initial screening, twelve articles were selected for data extraction. During data extraction, one additional article was excluded due to it not being published in English. The final review included eleven studies, nine from the database search and two selected from Google Scholar (Figure 1).

Across the eleven included studies, ultrasonic irrigation was evaluated for its potential to enhance root canal treatment outcomes compared with traditional syringe irrigation methods. The variation in the study protocol is presented in Table 3. Three main themes emerged from the included studies: microbial reduction, the influence of the irrigation protocol on post-operative pain, and the mechanistic basis of these improvements (Table 4).

## 4. Microbial Reduction

Across the included studies, incorporating ultrasonic irrigation into endodontic therapy consistently yielded greater bacterial reduction than conventional needle irrigation. One study reported a 47.22% reduction in microbial counts with ultrasonic irrigation compared with 37.97% with conventional syringe irrigation [21]. Another found a higher proportion of bacteria-free samples with ultrasonic irrigation, reducing positive bacterial cultures by 57% compared with 22.5% using only syringe for irrigation [26]. Ultrasonic activation of 17% ethylenediaminetetraacetic acid (EDTA) after chemomechanical preparation significantly enhanced endotoxin reduction in necrotic teeth with apical periodontitis (*p* < 0.05) [20]. Ultrasonic activation also produced substantially greater reductions in cultivable bacteria than conventional syringe irrigation (CNI)—a 98.4% median reduction vs. 23.6% (*p* < 0.05)—and rendered 80% of canals culture-negative compared with 30% for CNI [25].

## 5. Post Operative Pain

Findings on postoperative pain were mixed when comparing syringe irrigation with ultrasonic and other activation methods. Conventional syringe irrigation was associated with greater pain at 6 h than Endoactivator and ultrasonic approaches, with no significant differences thereafter [22]. Similarly, ultrasonic irrigation significantly reduced pain at 6 and 12 h compared with conventional syringe irrigation (*p* < 0.05), with no differences at 24 or 48 h [26]. Both PUA and laser-activated irrigation lowered pain scores at all measured time-points relative to CNI (*p* < 0.05), and analgesic consumption was highest with CNI, intermediate with laser activation, and lowest with PUA (*p* < 0.05) [23]. In a four-arm comparison of laser activation, photon-induced photoacoustic streaming (PIPS), PUA, and conventional irrigation, overall differences were significant (*p* < 0.05); post hoc testing showed the greatest pain reduction with laser activation, followed by PIPS, PUA, and then CNI [24]. Self-assessed pain at 24 h was also lower with ultrasonic than with syringe irrigation, with fewer acute postoperative reactions [28]. By contrast, several studies reported no significant differences in pain incidence between syringe and ultrasonic irrigation [18,19,27].

## 6. Mechanisms Explaining Improved Outcomes

Proposed mechanisms for the superior performance of ultrasonic irrigation include improved penetration and renewal of EDTA throughout the canal system, including uninstrumented recesses, driven by vigorous fluid movement along canal walls and increased contact with endotoxins [20]. Ultrasonic energy also induces acoustic streaming, cavitation, and mild warming of the irrigant, collectively extending antimicrobial reach into anatomically complex regions [25,26]. Passive ultrasonic (PUA) activation generates high-velocity irrigant flow that facilitates removal of debris from irregularities and oval canals [21]. These hydrodynamic effects support biofilm disruption, debris removal, tissue dissolution, and smear-layer elimination, which in turn improve lateral canal obturation and bacterial clearance [19].

Chemical and thermal contributions may further enhance outcomes: agitation of sodium hypochlorite (NaOCl) can elevate temperature and accelerate interactions with hard and soft tissues, promoting smear-layer dissolution [22]. Insufficient debridement with syringe irrigation may help explain higher postoperative pain scores [23]. Finally, oscillatory motion in ultrasonic systems tends to direct irrigant laterally toward canal walls, whereas syringe-based CNI preferentially drives irrigant apically, increasing the risk of apical extrusion and postoperative pain [24].

## 7. Discussion

In the context of this review, the outcomes investigated when comparing ultrasonic and syringe irrigation were microbial reduction and post-operative pain. Ultrasonic irrigation achieves greater reductions in endotoxin levels and cultivable bacteria than syringe irrigation [20,25]. Some trials show lower early postoperative pain with ultrasonic or sonic activation [22,23,24,26,28], though others report no significant difference [18,19,27]. Enhanced cleaning is attributed to improved irrigant penetration, acoustic streaming, cavitation, and heat-boosted chemical activity, which together disrupt biofilms and improve disinfection [20,21,26].

These results align with and extend prior literature suggesting superior canal cleanliness with ultrasonic activation compared with conventional syringe irrigation [11,29]. However, much of the earlier evidence derives from in vitro experiments, limiting direct clinical inference [11,29]. Consistent with this, the AAE notes better cleanliness with ultrasonic methods but a shortage of high-level clinical evidence directly linking activation to improved patient outcomes [30]. By synthesizing in vivo findings, the present scoping review begins to address this gap.

Although the included studies differed in instrumentation techniques and the types of teeth treated, these variables were controlled within each study. Leaving the irrigation protocol, ultrasonic activation and conventional needle irrigation, as the primary independent variables. While such factors are important to root canal prognosis, our review focuses on identifying potential trends in outcomes associated with ultrasonic irrigation.

### 7.1. Implications

The prognosis of root canal treatment remains the most critical indicator of long-term success. While the present review did not evaluate long-term clinical outcomes, it focused on bacterial reduction, a factor strongly correlated with favorable prognoses [31,32]. This finding highlights the importance of effective irrigation techniques in achieving microbial control, which is essential for treatment success. Although pain reduction does not directly affect the long-term prognosis, it represents an important clinical endpoint that contributes to patient satisfaction and overall treatment acceptance [33].

Given the anatomical complexity encountered in challenging endodontic cases, such as the presence of lateral canals, isthmuses, apical deltas, and severely curved or sclerosed root canals, effective disinfection remains a significant clinical challenge [34]. These intricate anatomical variations can harbor residual debris, necrotic tissue, and microorganisms that are often inaccessible through conventional instrumentation alone [35]. As a result, ultrasonic irrigation should be considered an essential adjunct in root canal therapy. Its ability to enhance the irrigant penetration and activation enables improved cleaning and disinfection of areas that are otherwise unreachable [36].

By integrating ultrasonic irrigation into the endodontic protocol, clinicians can potentially increase the likelihood of achieving a bacteria-free canal system, ultimately leading to improved treatment prognosis. Studies have demonstrated that ultrasonic irrigation accelerates and enhances the efficiency of canal debridement [37]. This enables clinicians to adopt more conservative mechanical shaping techniques, reducing the risk of complications associated with aggressive instrumentation, such as file separation, ledging, canal transportation, or perforation [38].

Consequently, ultrasonic irrigation not only supports minimally invasive endodontics but also contributes to enhanced safety and effectiveness. By reducing treatment time and improving disinfection, ultrasonic irrigation may decrease the likelihood of retreatment [4]. In addition, syringe irrigation is more likely to cause apical fluid extrusion due to the positive pressure applied during delivery [39]. These advantages are particularly meaningful for patients who travel long distances for care, as they help minimize chair time and reduce the need for follow-up appointments, thereby increasing convenience and overall treatment experience.

The advancement of ultrasonic irrigation technology has led to the development of more sophisticated systems, such as GentleWave [40]. Building on the principles of acoustic streaming and cavitation established by ultrasonic devices, GentleWave is designed to improve irrigant delivery via multisonic energy and negative pressure, aiming to enhance cleaning efficiency while preserving dentin integrity [41]. The search strategy of this review included the GentleWave system. However, no in vivo studies involving GentleWave were identified. As a relatively new innovation, first introduced in 2016, GentleWave remains underrepresented in the scientific literature. While early reports suggest promising results in terms of disinfection, there is limited high-quality evidence assessing its short- and long-term clinical efficacy [40,42]. Clinical trials are needed to determine whether GentleWave offers a significant advantage over established ultrasonic techniques, especially in complex cases or retreatment scenarios.

### 7.2. Limitations and Strengths

While this review provides a comprehensive summary of currently available studies involving human patients that compare ultrasonic and conventional irrigation techniques, several limitations must be acknowledged. First, major databases were searched; the number of databases was limited to four. This may have resulted in the exclusion of relevant studies not indexed in the selected databases. Additionally, English was the only common language among the reviewers, which led to the inclusion of only English-language publications. This linguistic limitation may have excluded pertinent research published in other languages.

Another key limitation is the small number of studies included in the final analysis. From an initial pool of 312 articles screened, only 11 met the inclusion criteria for in vivo studies, significantly constraining the dataset. This scarcity highlights a notable gap in the literature regarding clinical evaluations of ultrasonic irrigation in endodontics. Many studies on ultrasonic irrigation were excluded as they were conducted on extracted or bovine teeth. While these ex-vivo models are useful, they do not accurately represent the complexities of in-vivo conditions. Including such studies could have expanded the dataset but would have compromised the clinical applicability of the findings, as they provide limited insight into patient outcomes and treatment success.

It is important to acknowledge the substantial methodological heterogeneity across the included studies, particularly in terms of needle size and insertion depth, irrigation duration, the type and sequence of irrigants used, shaping technique, and initial diagnosis. These variables, along with procedural differences in chemical cleaning during instrumentation, may influence the reported efficacy of both conventional and ultrasonic irrigation methods. Additionally, postoperative pain is a multifactorial outcome that may be affected not only by the final irrigation approach but also by the overall shaping and irrigation protocols used throughout the procedure. Such methodological variations limit the ability to make direct comparisons between studies and may account for inconsistencies in reported outcomes.

This heterogeneity, especially in relation to postoperative pain, also limits the generalizability of our conclusions. While some studies suggest that ultrasonic irrigation reduces postoperative pain, others report no significant difference, reflecting inconsistency across the literature. These discrepancies may stem from differences in study design, patient populations, clinical techniques, and methods of pain assessment. As a result, it remains difficult to draw firm or universally applicable conclusions about the benefits of ultrasonic irrigation. This highlights the need for further well-designed, standardized studies to clarify its true impact on both treatment efficacy and patient-centered outcomes such as postoperative pain.

Despite these limitations, a major strength of this review lies in its exclusive focus on in vivo, human-based studies, which ensures greater clinical relevance and applicability to real-world endodontic practice. This approach allows for a more accurate assessment of ultrasonic irrigation’s clinical utility, setting this review apart from others that rely heavily on laboratory-based evidence.

### 7.3. Future Directions

Future research should prioritize clinical trials to address the current gap in in vivo evidence. The limited number of eligible clinical studies identified in this review underscores a critical need for high-quality research evaluating the real-world efficacy, safety, and long-term outcomes of ultrasonic irrigation in root canal treatment (RCT). Large-scale randomized clinical trials are essential to assess the comparative effectiveness of ultrasonic irrigation versus conventional irrigation across diverse patient populations and clinical scenarios. Particular attention should be given to complex cases, such as those involving severe canal curvature, calcifications, or anatomical anomalies, to determine the technology’s performance under more challenging conditions.

A key area for future investigation is the use of ultrasonic irrigation in retreatment cases. Research should evaluate its efficacy in removing gutta-percha, sealer remnants, and persistent biofilm from previously treated canals, which often present unique anatomical and microbial challenges. Furthermore, comparative studies between ultrasonic irrigation and newer technologies such as GentleWave are warranted to determine whether these innovations provide significant advantages in cleaning efficiency, dentin preservation, and overall treatment outcomes. Additionally, future research should aim to define optimal parameters for ultrasonic activation, including frequency, power output, and irrigant combinations. Establishing evidence-based protocols would help maximize effectiveness while minimizing potential risks, such as dentin damage or irrigant extrusion.

Long-term outcome studies are also needed to evaluate treatment success, including rates of periapical healing and tooth survival rates. Incorporating patient-reported outcomes, such as post-operative pain levels and satisfaction, will enhance the clinical relevance of future findings and support evidence-based decision-making.

## 8. Conclusions

This scoping review suggests that ultrasonic irrigation may improve root canal treatment outcomes in clinical settings when compared to conventional syringe irrigation. Through mechanisms such as acoustic streaming and cavitation, ultrasonic activation enhances disinfection, improves irrigant penetration, and increases procedural efficiency. The inconsistent evidence regarding post-operative pain highlights the need for larger, standardized studies to better define the true impact of ultrasonic irrigation on post-operative pain outcomes. Despite the limited number of in vivo studies, the available evidence supports the integration of ultrasonic irrigation into endodontic protocols to improve clinical outcomes and patient care.

## Figures and Tables

**Table 1:** Database search strategies employed.

Database (Date of Search)	Search Strategies	Number of Articles Found
PubMed (18 August 2025)	(“root canal” [tiab] OR “root canal therapy” [mesh] OR pulpectomy [tiab]) AND (acoustic [tiab] OR ultrasonic [tiab] OR multisonic [tiab] OR gentlewave [tiab]) AND (“side vented needle” [tiab] OR “syringe” [tiab] OR “conventional techniq*” [tiab] OR “conventional irriga*” [tiab]) AND (“efficacy” [tiab] OR “outcome*” [tiab] OR “failure” [tiab] OR “success” [tiab] OR “clinical outcome*” [tiab] OR “compar*” [tiab] OR “bacteria reduc*” [tiab] OR “post op pain” [tiab] OR “post operative pain” [tiab] OR “longevity” [tiab])	184
Scopus (18 August 2025)	TITLE-ABS-KEY ((“root canal” OR “root canal therapy” OR “pulpectomy”) AND (“acoustic” OR “ultrasonic” OR “multisonic” OR “gentlewave”) AND (“side vented needle” OR “syringe” OR “conventional techniq*” OR “conventional irriga*”) AND (“efficacy” OR “outcome*” OR “failure” OR “success” OR “clinical outcome*” OR “compar*” OR “bacteria reduc*” OR “post op pain” OR “post operative pain” OR “longevity”))	256
Dentistry and Oral Sciences Source (18 August 2025)	(AB (“root canal” OR SU “root canal treatment” OR “pulpectomy”) AND (“acoustic” OR “ultrasonic” OR “multisonic” OR “gentlewave”) AND (“side vented needle” OR “syringe” OR “conventional techniq*” OR “conventional irriga*”) AND (“efficacy” OR “outcome*” OR “failure” OR “success” OR “clinical outcome*” OR “compar*” OR “bacteria reduc*” OR “post op pain” OR “post operative pain” OR “longevity”)) OR (TI (“root canal” OR SU “root canal treatment” OR “pulpectomy”) AND (“acoustic” OR “ultrasonic” OR “multisonic” OR “gentlewave”) AND (“side vented needle” OR “syringe” OR “conventional techniq*” OR “conventional irriga*”) AND (“efficacy” OR “outcome*” OR “failure” OR “success” OR “clinical outcome*” OR “compar*” OR “bacteria reduc*” OR “post op pain” OR “post operative pain” OR “longevity”))	112
Google (18 August 2025)	“Ultrasonic Activation EDTA” AND “passive ultrasonic activation randomized clinical trial”	2

**Table 2:** Inclusion and Exclusion criteria employed during the data screening.

Inclusion Criteria	Exclusion Criteria
Published peer reviewed articles in English Research articles from 2015 to 2025 Study conducted in human subjects Focused on comparing conventional syringe irrigation with ultrasonic irrigation	Full text not available Review articles (literature review, narrative review, scoping review, systematic review, meta-analysis) Editorials Letters to the Editor Conference Proceedings/Conference Abstracts Studies Performed on Extracted Teeth Studies Performed on Bovine Teeth

**Table 3:** Summary of Study Design.

Author (Year)	Tooth Type/Diagnosis	Sample Size (Teeth)	Irrigation Methods Compared	Insrumentation Protocol of the Study	Ultrasonic Irrigation Protocol	CNI Protocol	Irrigant Concentrations	Deposition Distances from Working Length Activation Frequency
Ali (2023) [18]	Mandibular first molars Symptomatic irreversible pulpitis	78	XP Endo Finisher UltraX (Ultrasonic) Side Vented Needle	ProTaper Next (PTN) instruments were used to mechanically prepare the root canals in a crown-down technique using an endodontic motor with an adjusted torque of 2 Ncm and speed of 300 rpm according to the manufacturer’s instructions. The PTN rotary system was used as follows: X1, X2, followed by X3 as the master apical file. All files were used in a pecking motion.	3 mL of 2.5% NaOCl solution was passively introduced in each canal, Ultra X at the maximum power of 45 kHz with flexible and soft silver tip (21 mm, size 20 taper 2) was fitted passively reaching 2 mm short from the working length in short vertical strokes. The irrigation solution was activated for 1 min in each canal.	3 mL of 2.5% NaOCl solution was passively introduced in each canal by a side-vented needle that was passively placed at 2 mm short from the working length and was constantly pulsed in 1–2 mm vertical strokes for 1 min in each canal.	2.5% NaOCL	2 mm Short of Working Length 45 kHz
Chen (2016) [19]	Mandibular premolars with a single straight canal Periapical Periodontitis	60	Syringe Irrigation Ultrasonic Irrigation	Series of K-files using the step-back enlargement technique. The master apical file, which was three sizes larger than the initial apical file, was used for each pre-molar.	40 mL of 2.5% NaOCl and Odontonson-M ultrasonic instruments. #15 ultrasonic file was placed at the border between the lower third and middle third of the root canals. The oscillation was performed in bucco-lingual direction for 2 min without the ultrasonic file binding to the canals walls. The 2.5% NaOCl solution was delivered at a rate of 20 mL/min through the ultrasonic file.	(40 mL) of 2.5% NaOCl solution using a 5 mL syringe and a 27-gauge needle. The tip of the needle was placed at the border between the lower third and middle third of the root canals.	2.5% NaOCl	Border of Lower and Middle Third Manufacturers Reccomended Power Setting
Herrera (2017) [20]	Maxillary single-rooted teeth with one root canal per root Pulp necrosis and apical periodontitis	24	EDTA with Ultrasonic Activation EDTA Flushing	The cervical and middle thirds of the root canals were prepared with the crown-down technique using Gates Glidden burs. A size 10 K-file was used to obtain the full length of the root canal, using an apex locator. Next, root canals were instrumented using the Mtwo rotary nickel-titanium system according to the manufacturer’s instructions. The instrumentation sequence was as follows: size 10, 0.04 taper, size 15, 0.05 taper, size 20, 0.06 taper, size 25, 0.06 taper, size 30, 0.05 taper, size 35, 0.04 taper and size 40, 0.04 taper.	The root canal was flooded with 1 mL of EDTA and immediately activated for 30 s using an ultrasonic tip that was inserted 2 mm short of the root canal length. The power setting of the ultrasonic device was 30%); then, EDTA was aspirated and refreshed repeating the procedure twice.	The root canal was flooded with 1 mL of EDTA for 30 s; then, EDTA was aspirated and refreshed, repeating the procedure twice. Finally, the root canals were irrigated with 5 mL of saline.	2.5% NaOCl 2% Chlorohexidine 17% EDTA	2 mm Short of Working Length 30% Power of Ultrasonic Device
Jambagi (2021) [21]	Maxillary and mandibular single-rooted canals (permanent incisors, laterals, canines, premolars) Non-Vital	60	High Power Diode Laser Ultrasonic Irrigation Syringe (26 gauge)	ProTaper Universal rotary system using the crown down technique.	The root canal was ultrasonically irrigated (P5 Booster) for 3 min with a continuous flow of 50 mL with 2.5% NaOCl.	2 mL of 2.5% NaOCl, after each instrumentation with a disposable syringe of 26 gauge needle.	2.5% NaOCL 17% EDTA	Not Stated
Kathiria (2024) [22]	Permanent maxillary and mandibular molars Asymptomatic irreversible pulpitis	150	Side Vented Needle EndoActivator UltraX (Ultrasonic)	Canals were prepared with F-One single file system in crown down technique according to the manufacturer’s instruction, i.e., 2.5 controlled torque and at 500 RPM with X Smart EndoMotor. Orifice enlargement followed by cleaning and shaping was performed using 17/12 and 25/06 rotary files, respectively. Apical diameter was prepared up to #25 file.	2 mL of 3% NaOCl was flushed in the canal and irrigated using ultrasonic tip (Ultra X) at 2–3 mm to the working length. The solution was activated by vertical up and down strokes for 20 s. Repeat the cycle for 3–4 times.	4 mL of 3% NaOCl was flushed into all the canals using a side-vented 30-Gauge needle, 2 mm short from working length.	3% NaOCl 17% EDTA	2–3 mm Short of the Working Length Not Stated
Mathevanan (2023) [23]	Mandibular first molars Symptomatic irreversible pulpitis	75	Conventional Needle Irrigation Passive Ultrasonic Irrigation Laser Activated Irrigation	The glide path preparation was performed using continuous rotary instrumentation. All the canals were prepared using a continuous rotary system (ProTaper Gold).	Irrigants were dispensed into the canal using a 31G single port side vented needle and the final irrigant activation was performed using IRR 25/25 IrriSafeTM file driven by a P5 piezoelectric ultrasonic unit at a power setting of 5 while placing 2 mm short of working length. 3 mL of 3% NaOCl was used for each activation cycle for 30 s for a total of two cycles. and 3 mL 17% EDTA was activated for 30 s for two cycles followed by a final rinse of 3 mL of physiological saline to neutralize the effects of EDTA.	27 mm single side-port needle, 3 mL of 3% NaOCl was delivered subsequently into each prepared canal such that the needle was not bound and was placed 2 mm short of the working length and moved up and down with 2 mm amplitude for a total time of 30 s. An intermittent flush of 2 mL of physiological saline was carried out after the usage of NaOCl. 3 mL of 17% ethylenediaminetetraacetic acid (EDTA) was used as a final rinse for 1 min followed by irrigation using 5 mL of physiological saline to neutralize the effects of EDTA.	3% NaOCl 17% EDTA	2 mm Short of the Working Length Power Setting 5
Mittal (2023) [24]	Maxillary and mandibular molars Symptomatic irreversible pulpitis	60	New laser irrigation activation system shock wave-enhanced emission photoacoustic streaming (SWEEPS) Photon-induced photoacoustic streaming (PIPS) Passive ultrasonic irrigation activation techniques Conventional irrigation (CI) method.	Hyflex EDM files were used for root canal preparation according to the manufacturer’s instructions with canalPro endomotor.	Irrigation solution was activated using an ultrasonic tip (Ultra X) to an ultrasonic device (Ultra X). The activation of the tip was activated for around three times, with each cycle for 20 s along involving the use of 1 mL of 3% NaOCl. 2 mL of 17% EDTA solution was then activated for 1 min. Without touching the canal walls, the ultrasonic tip was placed 2 mm short of the working length of canal.	2 mL of saline with 2% povidone–iodine was used as the initial irrigant and this was followed by the NaOCl, and EDTA, as mid–rinses using 1 mL for each canal, followed by 2 mL of 2% chlorhexidine as last irrigant using 29–G side vented irrigation needle. The process of recapitulation was performed at each file change with a size #10 K–file.	10% EDTA 15% Carbamide Peroxide 2% Provodone Iodine 3% NaOCl 2% Chlorohexidine	2 mm Short of the Working Length Not Stated
Orozco (2020) [25]	Upper and lower single rooted teeth Pulp necrosis with radiographic evidence of apical periodontitis and intact pulp chamber walls	20	Passive Ultrasonic Activation Conventional Needle Irrigation	Instrumentation was performed by one single operator using single-file reciprocation technique. Initial apical instrument was ISO size #20 hand file, which reached passively to working length. The file was adapted to an electric motor using preset adjustments. The instrument was introduced into the root canal until resistance was felt and then activated. Next, the instrument was apically moved using in-and-out pecking motions, with approximately 3 mm in amplitude by using light apical pressure. After 3 pecking motions, the instrument was removed and cleaned. Between each third (cervical, middle, and apical), 8 mL of 2.5% NaOCl was used to neutralize the content inside the root canal. The working length (−1 mm) was determined by using an apex locator and confirmed by a periapical digital radiograph. Likewise, apical debridement was performed with a K-file size #30, which was extended 1 mm beyond this area. The root canal instrumentation was completed in a single visit in all cases, with a total of 24 mL of 2.5% NaOCl in both groups.	The root canals were irrigated with 4 mL of 2.5% NaOCl delivered by using a 31 gauge × 27 mm side port needle inserted up to 1 mm short of the WL, with PUA being performed for 30 s. The irrigating solution was renewed with 4 mL of 2.5% NaOCl and PUA was resumed for 30 additional seconds. For inactivation of 2.5% NaOCl, the canal was irrigated with 5 mL of 5% sodium thiosulfate, followed by irrigation with 10 mL of saline solution. The ultrasonic activation was performed with a #20:01 non-cutting tip (E1 Irrisonic) and piezoelectric ultrasonic device (ALT—Equipamentos Médicos e Odontológicos) at 1000 Hz low power. The ultrasonic instrument was used at 1 mm short of the WL, avoiding contact with the root canal walls.	The root canals were irrigated with 8 mL of 2.5% NaOCl by using a 31 gauge × 27 mm side port needle, inserted up to 1 mm short of the WL, and 17% EDTA remained inside the root canal for 4 min and manually agitated for 1 additional minute. The 2.5% NaOCl inactivation and 17% EDTA removal were performed in the same manner as described for PUA group. No ultrasonic activation was performed in this group.	2.5% NaOCL 17% EDTA	1 mm Short of the Working Length 1000 Hz
Palanisamy (2023) [26]	Single–rooted teeth Pulpal necrosis	80	Passive Ultrasonic Irrigation Side Vented Needle Irrigation	Canal instrumentation was accomplished using rotary and hand files, as well as 5.25% sodium hypochlorite irrigation. All teeth were prepared with ProTaper rotary instruments.	Agitated with a 150 μm, noncutting, stainless steel wire (Irrisafe) attached to an ultrasonic device (P5 Newtron unit) set to 10 (frequency, 30 kHz). The irrigation protocol was similar to that used in Group CNI, but the irrigants were passively agitated using Irrisafe tips (#15 ultrasonic file) for 30 s before changing the solution.	6 mL of 2.5% NaOCl and a 31G, 27 mm NaviTip Sideport needle, 1 mm from the working length for 3 min (flow rate, 2 mL/min). This was followed by irrigation with 1 mL of 17% EDTA for 1 min and 1 mL of 2% chlorhexidine for 1 min.	2.5% NaOCL 17% EDTA 2% Chlorohexidine	1 mm Short of the Working Length 30 kHz
Parvez (2022) [27]	Permanent molars Asymptomatic irreversible pulpitis, periapical periodontitis	120	Side Vented Needle Passive Ultrasonic Irrigation	Group 1: The canal was cleaned and shaped by hand, using K-files and the step-back technique. Group 2: The ProTaper Next crown down technique was used for cleaning and shaping. Glyde was used to lubricate the canals during the preparation process. Preparation began with X1, then X2, then X3, with apical rotation at 300 rpm and torque of 2–5.2 N/s using the X smart plus endo-motor. Group 3: Cleaning and shaping were carried out with Wave One Gold reciprocating files using gentle inward motion. During preparation, canals were lubricated with glyde. Withdrawing the file every 3 mm to remove debris. Shaping was achieved at the definitive working length with X smart plus endomotor.	Group 1B: Irrigation with 2.5 percent NaOCl and 17 percent EDTA was performed during the preparation, the canals were kept filled with 2.5 percent NaOCl, and the ultrasonic file (Mani Inc.) was kept short by 2 mm from the working length and free from the canals before passive ultrasonic irrigation was performed for 1 min. Group 2B: Irrigation with 2.5 percent NaOCl and 17 percent EDTA was performed during the preparation, the canal was kept filled with 2.5 percent NaOCl, and the ultrasonic file was kept short by 2 mm from the working length and free from the canals, then passive ultrasonic irrigation was performed for 1 min. Group 3B: Irrigation with 2.5 percent NaOCl and 17 percent EDTA was performed during the preparation. The canal was kept filled with 2.5 percent NaOCl and the ultrasonic file was kept short by 2 mm from the working length and free from the canals, after which passive ultrasonic irrigation was performed for 1 min.	Group 1A: Irrigation was performed with 2.5 percent NaOCl using a 27 gauge side vented needle (Acteon) and 17 percent EDTA during the preparation, followed by final irrigation with normal saline (0.9% *w*/*v*). Group 2A: Irrigation with 2.5 percent NaOCl and 17 percent EDTA was performed throughout the preparation, and final irrigation was done with normal saline using a 27 gauge side vented needle. Group 3A: During the preparation, irrigation was done with 2.5% NaOCl using a 27 gauge side vented needle and with 17% EDTA, final irrigation was carried out with normal saline.	2.5% NaOCL 17% EDTA	2 mm Short of the Working Length Not Stated
Tang (2015) [28]	Incisors, Premolars, Molars Periapical Periodontitis	360	Ultrasonic Irrigation Syringe Irrigation	Mtwo NiTi files were used for the canal preparation.	Group A: 2.5% sodium hypochlorite solution in ultrasonic irrigation. Group B: Active silver ion antibacterial solution in ultrasonic irrigation.	Syringe irrigation with 2.5% sodium hypochlorite solution.	2.5% NaOCl	Not Stated

**Table 4:** Summary of Study Findings.

Author (Year)	Irrigation Methods Compared	Comparison in Bacterial/Endotoxin Reduction	Pain Scales Utilized	Comparison of Post Operative Pain	Proposed Mechanisms
Ali (2023) [18]	XP Endo Finisher UltraX (Ultrasonic) Side Vented Needle	Not Applicable	Verbal Rating Scale	No statistically significant difference was found between all groups regarding the incidence and intensity of pain at different time intervals (*p* > 0.05).	Not Applicable
Chen (2016) [19]	Syringe Irrigation Ultrasonic Irrigation	Not Applicable	Incidence of Pain	No significant difference in the incidence of pain was observed between the syringe irrigation and ultrasonic irrigation groups.	Not Applicable
Herrera (2017) [20]	EDTA with Ultrasonic Activation EDTA Flushing	A statistically significant difference was found in the median percentage values for the reduction in cultivable bacteria (*p* < 0.05) between CNI (23.56%) and PUA (98.37%), producing 30% and 80% root canals free of cultivable bacteria in CNI and PUA group, respectively, in endodontic treatment The number of cultivable bacteria significantly decreased in PUA group, with 98.37% reduction percentage when comparing with CNI group, which only reduced 23.56%.	Not Applicable	Not Applicable	The energy transmitted might lead to an acoustic streaming, cavitation, and/or warming of the irrigating substance, expanding its spectrum of action, especially on microorganisms in difficult-to-reach areas.
Jambagi (2021) [21]	High Power Diode Laser Ultrasonic Irrigation Syringe (26 gauge)	Highest reduction in the microbial count was seen in diode laser group (60.92%), ultrasonic group was second with (47.22%), and least reduction was observed in conventional irrigation (37.97%).	Not Applicable	Not Applicable	Flow of the irrigant at a high velocity which was achieved due to passive ultrasonic activation thus facilitating the removal of debrisfrom the root canal irregularities and oval-shaped canals.
Kathiria (2024) [22]	Side Vented Needle EndoActivator UltraX (Ultrasonic)	Not Applicable	Visual Analog Scale	Side vented needle had more pain when compared with Endoactivator and Ultrasonic groups at 6 h, but there was no statistically significant difference in pain after the intial 6 h.	Damage to microorganisms and physical destruction of biofilm is the result of explosive and implosion, which is created by sheer stress. This might be the reason for less pain after 6 h of follow-up with UX.
Mathevanan (2023) [23]	Conventional Needle Irrigation Passive Ultrasonic Irrigation Laser Activated Irrigation	Not Applicable	Visual Analog Scale	The pairwise comparison showed that PUI and LAI group had a significant reduction in pain scores at all experimental periods when compared to CNI (*p* < 0.05), with no significant difference in PUI and LAI in postoperative pain scores at all the assessed time intervals. The consumption of analgesic was assessed and participants who received CNI were subjected to more incidence of analgesic consumption followed by LAI and PUI, respectively (*p* < 0.05).	Insufficient pulpal debridement with CNI leading to more post-operative pain scores
Mittal (2023) [24]	New laser irrigation activation system shock wave-enhanced emission photoacoustic streaming (SWEEPS) Photon-induced photoacoustic streaming (PIPS) Passive ultrasonic irrigation activation techniques Conventional irrigation (CI) method.	Not Applicable	Visual Analog Scale	Significant difference (*p* < 0.05) between all study groups. Post hoc statistical analysis revealed that pain score decreased significantly in Group 4 (SWEEPS), followed by Group 3 (PIPS), Group 2 (ultrasonic activation), and Group 1 (conventional needle irrigation)	The oscillatory movement in ultrasonic system pushes the irrigants laterally to the canal walls, whereas with CI with syringe, irrigants constantly move apically. This causes irrigants to extrude apically, leading to increased incidence of postoperative pain.
Orozco (2020) [25]	Passive Ultrasonic Activation Conventional Needle Irrigation	A statistically significant difference was found in the median percentage values for the reduction in cultivable bacteria (*p* < 0.05) between CNI (23.56%) and PUA (98.37%), producing 30% and 80% root canals free of cultivable bacteria in CNI and PUA group, respectively, in endodontic treatment The number of cultivable bacteria significantly decreased in PUA group, with 98.37% reduction percentage when comparing with CNI group, which only reduced 23.56%.	Not Applicable	Not Applicable	The energy transmitted might lead to an acoustic streaming, cavitation, and/or warming of the irrigating substance, expanding its spectrum of action, especially on microorganisms in difficult-to-reach areas.
Palanisamy (2023) [26]	Passive Ultrasonic Irrigation Side Vented Needle Irrigation	Ultrasonic irrigation had a higher proportion of bacteria-free samples than Side Vented Needle irrigation. The passive ultrasonic irrigation group reduced positive bacterial culture by 57%, while the side-vented needle irrigation group reduced it by 22.5%	Visual Analog Scale	Ultrasonic showed a significant reduction in pain at 6 and 12 h compared to side vented needle (*p* < 0.05), but there were no significant differences observed at 24 and 48 h time-points.	Acoustic streaming and cavitation during passive ultrasonic irrigation.
Parvez (2022) [27]	Side Vented Needle Passive Ultrasonic Irrigation	Not Applicable	Numeric Pain Scale	The use of a needle versus passive ultrasonic irrigation had no statistically significant differences.	Not Applicable
Tang (2015) [28]	Ultrasonic Irrigation Syringe Irrigation	Not Applicable	Visual Analog Scale	Self-assessed pain levels 24 h after the procedure are significantly lower for Ultrasonic than for Syringe (*p* < 0.05). Ultrasonic showed lower incidences of postoperative acute reactions.	Not Applicable
